# Phenomenology and therapeutic potential of patient experiences during oral esketamine treatment for treatment-resistant depression: an interpretative phenomenological study

**DOI:** 10.1007/s00213-023-06388-6

**Published:** 2023-05-24

**Authors:** Joost J. Breeksema, Alistair Niemeijer, Bouwe Kuin, Jolien Veraart, Eric Vermetten, Jeanine Kamphuis, Wim van den Brink, Robert Schoevers

**Affiliations:** 1 Department of Psychiatry, Research School of Behavioural and Cognitive Neurosciences (BCN), University Medical Center Groningen, University of Groningen, Postbus 30.001, 9700 RB Groningen, the Netherlands; 2 Department of Care Ethics, University of Humanistic Studies, Utrecht, The Netherlands; 3 PsyQ Haaglanden, Parnassia Psychiatric Institute, The Hague, the Netherlands; 4 Amsterdam UMC, location Department of Psychiatry, Academic Medical Center, Amsterdam, the Netherlands; 5 Program Compulsivity, Impulsivity & Attention, Amsterdam Neuroscience Research, Amsterdam, the Netherlands; 6 Department of Psychiatry, Leiden University Medical Center, Leiden, the Netherlands

**Keywords:** Esketamine, Phenomenology, Psychotherapy, Treatment-resistant depression, Patient experiences, Qualitative research

## Abstract

**Background:**

Ketamine and its enantiomers are widely researched and increasingly used to treat mental disorders, especially treatment-resistant depression. The phenomenology of ketamine-induced experiences and their relation to its psychotherapeutic potential have not yet been systematically investigated.

**Aims:**

To describe the phenomenology of patient experiences during oral esketamine treatment for treatment-resistant depression (TRD) and to explore the potential therapeutic relevance of these experiences.

**Methods:**

In-depth interviews were conducted with 17 patients after a 6-week, twice-weekly ‘off label’ generic oral esketamine (0.5–3.0mg/kg) treatment program. Interviews explored participants’ perspectives, expectations, and experiences with oral esketamine treatment. Audio interviews were transcribed and analyzed using an Interpretative Phenomenological Analysis (IPA) framework.

**Results:**

The effects of ketamine were highly variable, and psychological distress was common in most patients. Key themes included (a) perceptual effects (auditory, visual, proprioceptive), (b) detachment (from body, self, emotions, and the world), (c) stillness and openness, (d) mystical-type effects (transcendence, relativeness, spirituality), and (e) fear and anxiety. Key themes related to post-session reports included (a) feeling hungover and fatigued, and (b) lifting the blanket: neutralizing mood effects.

**Conclusion:**

Patients reported several esketamine effects with psychotherapeutic potential, such as increased openness, detachment, an interruption of negativity, and mystical-type experiences. These experiences deserve to be explored further to enhance treatment outcomes in patients with TRD. Given the frequency and severity of the perceived distress, we identify a need for additional support in all stages of esketamine treatment.

## Introduction

Research into the antidepressant potential of ketamine has proliferated since the early 2000s. The potential therapeutic role of this compound in psychiatry has been explored since the early 1970s (Khorramzadeh and Lotfy, 1973). Its psychoactive effects include visual and auditory perceptual changes, altered proprioception, alterations in consciousness, detachment from the world or self, and occasionally ‘mystical’ experiences (Mollaahmetoglu et al., 2021; Sumner et al., 2021; van Schalkwyk et al., 2017). In mental health contexts, these effects have alternately been labeled ‘dissociative’ (Corssen and Domino, 1966; Domino et al., 1965), psychotomimetic (mimicking a psychotic state) (Krystal et al., 1994), or ‘psychedelic’ (Mashour, 2022).

Presently, ketamine and its enantiomers (hereafter: ‘ketamine’) are mainly administered as a regular pharmacological treatment without explicit psychological support or psychotherapy, and its psychoactive effects are often considered undesirable side effects (Grabski et al., 2020; Luckenbaugh et al., 2014; Mathai et al., 2020). Some therapists integrate ketamine administrations in a broader psychotherapeutic framework as ketamine-assisted psychotherapy (KAP) (Drozdz et al., 2022; Joneborg et al., 2022; Mathai et al., 2022a). However, approaches vary, no evidence-based treatments for KAP exist, and research on lived experiences and their relation to the therapeutic process is scarce.

Only a few recent qualitative studies have explored patients’ perspectives on ketamine treatment. Moreover, these studies have primarily involved intravenous racemic ketamine to treat (treatment-resistant) depression and suicidality, with one article describing treatment of patients with alcohol use disorder. In these studies, the following themes were identified: treatment enablers and obstacles, patient attitudes, (non)-recovery narratives, perceived adverse effects, and the phenomenology of the acute effects of ketamine, including perceptual alterations, emotional or mood changes, and cognitive effects (Griffiths et al., 2021; Jilka et al., 2021; Lapidos et al., 2023; Lascelles et al., 2019, 2021; Mollaahmetoglu et al., 2021; Sumner et al., 2021; van Schalkwyk et al., 2017). In 2019, an esketamine nasal spray was registered for treatment of treatment-resistant depression (TRD). In the European Union, esketamine has been registered as a generic anesthetic drug since 1997.

Like intravenous infusions, intranasal administration is presently only allowed to be administered under professional supervision in a psychiatric hospital and comes with significant financial costs, limiting accessibility. Oral administration of generic esketamine is less costly, less invasive for patients than most routes of administration, and in principle suitable for at-home treatment. To our no knowledge, no qualitative studies have explored patient experiences with orally administered esketamine. Patients’ experiences can inform us about helpful, less helpful, or negative treatment aspects, and can provide insights into potentially relevant psychological processes, which may help optimize treatment approaches for vulnerable patients. To investigate patients’ perspectives and experiences, we conducted a phenomenological study using in-depth qualitative interviews in patients with severe TRD receiving off-label treatment with a generic oral esketamine solution.

This study is part of a larger qualitative study aimed at studying all aspects of oral esketamine treatment in TRD patients, including phenomenological and non-pharmacological aspects. In an earlier publication on the same sample, we explored non-pharmacological facets, including the impact of set and setting on session experience, describing patients’ (in) ability to let go of control (Breeksema et al., 2022a). The primary aim of the current study is to describe the phenomenology of patient experiences during oral esketamine administration. Our secondary aim is to explore the potential therapeutic relevance of these experiences.

## Methods

### Design

This qualitative study is based on individual, in-depth interviews, conducted within an Interpretative Phenomenological Analysis (IPA) framework (Pietkiewicz and Smith, 2014; Smith, 2004; Smith and Osborn, 2007) in order to explore the experiences and perspectives of patients undergoing oral esketamine treatment for TRD. IPA is directed at the understanding of complex subjective phenomena by examining a subjects’ lived experience of a phenomenon in detail (Finlay, 2014; Miller and Barrio Minton, 2016). IPA emphasizes idiography, i.e., fully exploring individual stories before examining how these experiences converge and diverge within the target group. For a more extensive description of method and analysis, we refer to Breeksema et al. (2022b).

### Treatment setting and participants

All participants in this qualitative study were patients with TRD receiving individually titrated off-label generic oral esketamine injection fluid mixed in a liquid formulation. Doses started at 0.5 mg/kg and were titrated up in 0.5mg/kg steps to a maximum of 3.0 mg/kg based on tolerability and antidepressant effects. The 6-week, twice-weekly treatment was provided at two specialized depression clinics in the Netherlands. Patients were severely treatment resistant, with a mean number of 9.3 failed antidepressant interventions; 60% had also had electroconvulsive therapy (ECT) with insufficient response (see Table 1 for full demographic information). Mean duration of their current depressive episode was 55 months. Patients did not receive any preparatory and/or integrative psychotherapy related to the use of esketamine.

**Table 1:** Demographic and clinical characteristics of respondents

#	Sex	Age range	Diagnosis	Psychiatric comorbidity	Depression treatment history	# esketamine sessions
P1	F	60–65	MDD	PD-NOS	- Multiple psychotherapies - Multiple ADs (SSRIs, SNRIs, TCAs)	15
P2	M	30–35	MDD	-	-Multiple ADs (SSRI, SNRI, MAOI, lithium), bupropion -ECT, rTMS	11
P3	F	45–50	MDD	APD PTSD symptoms	-Multiple psychotherapies -Multiple ADs (SSRIs, SNRI, TCAs, mirtazapine, lithium, and quetiapine addition), bupropion -ECT	22
P4	F	35–40	BPD II	C-PTSD	-Multiple psychotherapies -Bupropion, multiple mood stabilizers	62
P5	M	55–60	MDD	ASS	-Multiple ADs (SSRIs, SNRIs, TCAs, MAOIs, lithium addition, antipsychotics addition) -ECT	22
P6	F	40–45	MDD	-	-Multiple ADs (SSRIs, SNRIs, TCAs), bupropion -Multiple psychotherapies	11
P7	M	55–60	MDD	-	-Multiple ADs (SSRIs, SNRIs, TCAs, lithium addition) -Multiple psychotherapies	12
P8	M	40–45	BPD I	-	-Multiple ADs (SSRIs, SNRIs, TCA, lithium addition) -Multiple psychotherapies	16
P9	F	60–65	MDD	-	-Multiple ADs (SSRIs, SNRIs, TCA + lithium addition)	23
P10	F	50–55	MDD	ASS, PTSD	-Multiple ADs (SSRIs, SNRIs, TCA, MAOIs, lithium addition, antipsychotics addition) - EMDR, ECT	24
P11	F	60–65	MDD	-	-Multiple ADs (SSRIs, SNRIs, TCAs, MAOIs, lithium addition) -Multiple psychotherapies -ECT	316
P12	M	55–60	MDD	-	-Multiple ADs (SSRIs, SNRIs, TCAs, MAOIs, mirtazapine, lithium addition, antipsychotics addition), topiramate -EMDR	90
P13	F	60–65	MDD	-	-Multiple ADs (SSRIs, SNRIs, TCAs, MAOIs, lithium addition, antipsychotics addition) -Multiple psychotherapies -ECT	350
P14	F	50–55	MDD	-	-Multiple ADs (SSRIs, SNRIs, TCAs, MAOIs, lithium addition, antipsychotics addition) -Multiple psychotherapies	~65
P15	M	40–45	BPD I	ADHD, migraine headaches	-Multiple ADs (SSRIs, SNRIs, TCAs, MAOIs, mirtazapine, lithium addition, antipsychotics addition) -ECT, light therapy	~45
P16	F	40–45	MDD	ASS	-Multiple ADs (SSRIs, SNRIs, TCAs, MAOIs, lithium addition, antipsychotics addition) -Multiple psychotherapies -ECT, IV esketamine	28
P17	F	60–65	MDD	-	-Multiple ADs (SSRIs, SNRIs, TCAs, MAOIs, lithium addition, antipsychotics addition) -Multiple psychotherapies	12

*ADs* antidepressants, *ADHD* attention deficit hyperactivity disorder, *APD* avoidant personality disorder, *ASS* autism spectrum disorder, *BPD* bipolar disorder, *EMDR* eye movement desensitization and reprocessing, *ECT *electroconvulsive therapy*, MAOis* monoamine oxidase inhibitors, *MDD* major depressive disorder, *PD-NOS* personality disorder not otherwise specified, *PTSD* post-traumatic stress disorder, *SNRIs* serotonin-norepinephrine reuptake inhibitors, *SSRIs* selective serotonin reuptake inhibitors, *TCAs* tricyclic antidepressants

Purposive sampling was used to recruit participants; we invited seventeen individuals (11 women and 6 men), all of whom accepted, participated in an interview, and provided written informed consent to audio record interviews and publish data, ensuring confidentiality and anonymity. The treatment protocol (M17.217644) and the qualitative research protocol (M20.256068) were submitted separately to the Medical Ethics Review Board of the University Medical Center Groningen (METc UMCG). The METc exempted both protocols from review, concluding that these do not constitute clinical research with human subjects as meant under the Dutch Medical Research involving Human Subjects Act (WMO) 1999, and that approval was therefore not needed. Off label treatment was approved for TRD and started in 2014 in our center. It should be noted that esketamine had already been a registered anesthetic medication in the Netherlands since 1997 (in contrast to, for example, the USA where esketamine is not registered for anesthesiology). Participants were informed that study participation was voluntary and would not affect their treatment. No compensation was offered.

### Data collection

A total of 17 in-depth interviews (14 by JJB, three by BK) were conducted. In order to capture the potential variability of effects over the 6-week treatment course, as well as explore patients’ experiences with and perspectives on the treatment as a whole, most interviews were conducted shortly after the initial 6-week treatment, i.e., after 12 esketamine sessions. Additionally, we included several participants who also had several months experience of at-home esketamine use. Interviewers were not involved in the treatment and had no prior contact with participants. Interviews followed a semi-structured interview guide, designed to inquire about participants’ experiences with esketamine sessions, and the treatment as a whole. The interview guide included open-ended questions about ‘acute’ experiences during the ketamine sessions (‘can you describe what happened during the ketamine sessions?’) and their experiences after the effects had worn off (‘how did you notice any impact of the treatment?’), intended to explore the phenomenology of treatment sessions, and attributions of meaning. For the full interview guide, see Breeksema, Niemeijer, et al. (2022a). Interviews lasted 82min on average. Most interviews took place online due to covid restrictions; three were conducted in-person. Data saturation, i.e., when it was felt that interviews yielded no new insights, was used to decide when to stop including new participants. Due to the phenomenological nature of the study, our focus is on the extent of variation and how exemplary lived experiences were, rather than numerical or statistical frequency (Corbin and Strauss, 2008), emphasizing theoretical generalization from statistical significance (Niemeijer, 2015).

### Data analysis

Transcribed interviews were analyzed with the support of qualitative data analysis software MAXQDA. We followed IPA (Pietkiewicz and Smith, 2014; Smith and Osborn, 2007, 2008) during the iterative analysis process, which draws from phenomenological, heuristic, and idiographic backgrounds, and concerns both the description and the interpretation of participants’ experiences. First, all transcripts were read and then analyzed individually. Comments were added, which were refined into themes of a higher (psychological) abstraction. These themes were re-examined, tentative patterns among themes identified, which were clustered based upon conceptual similarity and differentiation, resulting in a list of major themes and sub-themes. All authors read parts of transcripts; observations, themes, interpretations, and reflections were discussed between authors until consensus emerged. After individual case analyses, a within-group analysis was conducted, grouping themes based on conceptual similarities between individual participants, identifying both convergent and divergent topics (where patient experiences differed) [20]. Emerging themes were identified and tentatively organized. These themes were then categorized under higher-level categories, which were discussed with different authors. This way, all transcripts were discussed within the multidisciplinary research team until consensus emerged, to triangulate the data and ensure validity. The standards for reporting qualitative research (SRQR) and the consolidated criteria for reporting qualitative research checklists (COREQ) were followed to ensure maximal methodological rigor (O’Brien et al., 2014; Tong et al., 2007).

## Results

**Fig. 1:**
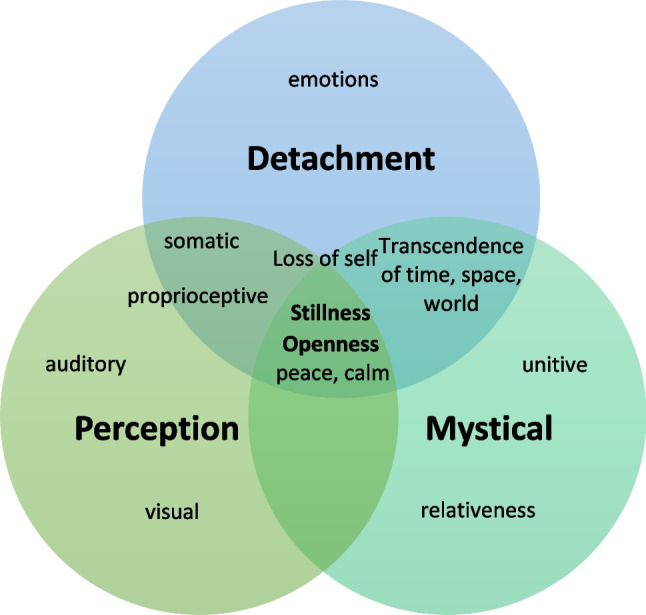
Overlapping phenomena described by patients during the acute phase of oral esketamine sessions

Table 2 lists the key themes and sub-themes that were identified, which are explored in more detail below. As seen in Fig. 1, these themes partially overlapped.

**Table 2:** Key themes, subthemes, and example citations

Acute (during esketamine administration)		
Theme	Subthemes	Example citations
Perceptual	Auditory	*“The music changes, it’s so weird, going from fast to very slow, slow songs become even slower. Everything changes, becoming at once very loud or very soft.” *(P15)
	Visual	*“It feels like [a trip]. Like my mind is detached from my body. It goes in all directions, so to say (…) a rollercoaster of colors, light, sounds, thoughts.” *(P11)
	Proprioceptive	*“I started feeling a vibration in my body, slowly and as it was getting worse it was as if I could hear it. A kind of humming or like [the buzzing sound of] your phone vibrating.”* (P7)
Detachment	From body	*“Suddenly, all at once, I enter this experience, I am taken along (…) I no longer have my body (…) I have no idea that I still have a body, so to speak, then it really only is my mind.” *(P4)
	From self	*“You don't know where you are. Are you just a little cell, or are you a particle, or…? Something, but not something with two hands, two legs and a head and a torso. (…) My self is just gone. These were really experiences outside myself.”* (P5)
	From emotions	*“When I’m deep in my depression (…) it feels as if my heart is literally breaking it hurts so much. (…) During the ketamine, my head is empty, but that deep feeling of depression that is not so much in my head, but in my body, is also gone. So that gives a lot of relief.”* (P17)
	From external world	*“You actually end up in a sort of different reality (…) [It is] a kind of waking up. [In the sense] that our daily life is a kind of dream state, a sort of program, an automatic thing (…) ketamine makes you aware of.”* (P7)
Tranquility and openness	Openness	*“The story of [me] as a patient lying here (…) completely disappeared. (…) Just a total openness (…) a kind of primal feeling which I cannot quite describe, a clear feeling of ‘this is it!’ This unity, where for a moment [my story] diminishes. Not as a thought, more as a kind of realization. And in a pleasant way.” *(P2)
	Peace, calm	*“When I am carried along and upwards towards the light [it is all] just peaceful, light, and it just feels just nice, and then I feel myself [floating] like a feather all the way up.”* (P4)
Mystical-type effects	Transcendence of time and space	*“I'm just really completely gone. (…) I don't perceive the outside world either. I'm not able to move. (…) I'm not here, on earth, anymore. I'm floating somewhere in that infinite world that doesn't end.”* (P10)
	Relativeness	*“You step outside of your particular little personhood; you get a kind of broader perspective (…) The sense of unity was very strong there (…) so the person you are necessarily does lose a bit of its importance.”* (P8)
	Unity	*“It's like the golden ratio, which recurs a lot in nature, in shells and so on, which comes back to perfection, which I actually experienced, the perfection of everything, a kind of unity.” *(P7)
	Spiritual/religious experiences	*“Suddenly, I heard - I can't say I heard a voice, but a phrase came into my head: dissolved in the All. (…) and an enormous sense of peace came over me.” *(P17)
Fear and anxiety		*“Sometimes I knew that the things I saw or heard were not really there. For example, I once thought that a blanket was a monster, or that I heard a scream outside. And although I was still able to think they were not real, it does feel real, and it did make me afraid ... and it’s hard work to stay with it.” *(P3)
Post-session		
Fatigue and hangover	Fatigue	*“It's a very heavy feeling, like if you've been running or exercising or really exerted yourself physically (…) Then everything is heavy, I can’t manage anymore, I need to take a rest for a while I need to come to myself (….) This lasts at least until the afternoon and the next day, it's usually over, although occasionally you still feel like that…” *(P14)
	Hangover	*“A side-effect that I could have on the evening of the session day. And often the day after as well (…) Feeling like crap. Hungover and just not feeling like anything. Not wanting to eat either. Wobbly and nauseous, dizzy, headache. Things like that.” *(P3)
Lifting the blanket	Mood effects	*“[When I started] I was really at the bottom of the depression, and that has gradually gotten better. And it’s now, say, between a 3 and a 6 [out of 10], and anyway, that's really different than being at 1 or 2. (…) But of course, it would be nice if that could go up a bit more, and that it would become a bit more stable.”* (P17)

### Acute (during session) experiences

#### Perceptual effects

Esketamine induced visual, auditory, and proprioceptive effects. A few participants described visual effects, including colorful spirals, geometrical patterns, or visual images reminiscent of science fiction movies. Several participants reported that environmental noises sounded as if they were sped up, slowed down, or intensified. Others described specific auditory effects: buzzing, crackling, whooshing, bubbling, or humming sounds.

*“A ticking heater (…) someone outside mowing the lawn… that hum becomes gigantic, really overwhelms everything.”* (P11)

The most pronounced and immersive perceptual effects were alterations in proprioception, or how participants experienced their own body. In the early phase of sessions, the experience was likened to a sensation of floating, falling, flying, or being on a rollercoaster. This experience was pleasant for some, but negative for others, who felt as if they were drowning or suffocating.

*“[It felt like] lying on a huge air mattress, which was deflated at certain places, so that I was sucked into one of those holes, that went empty while I was lying there.” *(P7)

#### Detachment

With increased intensity, these perceptual effects became more dissociative-like and detached: participants felt their body sink away, feeling as if their body (parts) no longer belonged to them, limbs seemingly becoming very large, small, or out of proportion. Some participants described becoming even more detached from their bodies, sometimes entirely until they were ‘only mind’.

*“It was like floating out of your body, going up and outside of my body, floating around freely without space and time. Lovely.” *(P16)

Particularly at higher dosages, several respondents described being drawn or sucked into a different reality, realm, or dimension.

*“You just get sucked into one point. And there's no escaping that. (…) You have no body, you're not a person. You are something else at that moment. (…) It’s strange. You don't know where you are. Are you just a cell, or are you a particle? A part of something? (…) My [self] is just gone. These were really experiences outside of myself.”* (P5)

Several respondents also described becoming detached from entrenched negative emotions or experiencing few(er) emotions during the esketamine sessions. Experiences could still be pleasant or unpleasant, but for most respondents, specific emotions did not appear to play a large role.

*“It is a physical experience, and indeed thoughts pass by, and the feeling that my mind is separate from my body and that it’s my mind experiencing it (…) but I have no emotions, not that I become happy or sad. It is really purely (…) physical and mental.”* (P11)

#### Tranquility and openness

Several participants noticed a detachment or interruption of their negativity, hopelessness, and despair. This transient absence of incessant negative thoughts was associated with an enlarged space and openness to other thoughts, ideas, and emotions. This emptiness or spaciousness was frightening to some, but many found this a positive sensation, using words like open, light, spacious, stillness, calm, relaxation, or peace to describe the experience.

*“My head became completely empty and completely calm, like a sort of numbness, a feeling like almost like floating (…) It felt very relaxing. (…) I know very well where I am, it was more of a pleasant not-being. Just away from the world, almost, but not frightening.”* (P17)

The temporary cessation of negative or suicidal thoughts also enabled some participants to distance themselves better from entrenched dark thoughts and ruminations.

*“The head is empty, and it's just so special after five years of being inside your head where it's all very nasty. That suddenly those thoughts and feelings disappear. It's very bizarre. But very pleasant. And of course, they do return, but during the buzz they were gone.”* (P17)

Paradoxically, as participants described becoming more detached from negative emotions, they also described having more room for emotions, both positive (joy, pleasure) and negative (sadness, grief). As they became more in touch with their own feelings and needs, some participants felt less self-critical or were able to put their own needs first.

*“I also notice that, because I get less depressed, the sadness comes more to the surface. And because of that I think I am also more receptive to therapy now.”* (P1)

This increased openness facilitated both connections to respondents’ emotions and their ability to connect to others; several respondents felt as if they had more space to be interested in family, friends, and loved ones, to reach out to them, and to make different choices based on the importance of interpersonal connections.

*“I do notice that I am a little more in touch with myself. I write and make poetry [again] (…) Also in contact with others, in order to let them know how I’m doing. (…) I notice that I [still] ignore or avoid things, but now I manage a little better to reach out and communicate that to others.”* (P3)

For some, having a positive experience or being lifted out of their depressive state for a few moments was intrinsically valuable.

*“It was very beautiful to have felt, to have experienced this. (…) I can still recall that feeling, even though it has been a few months (…) It doesn't have the same impact now, in terms of ridding me of those heavy thoughts and feelings, but I do try to hold on to that experience and keep it with me. (…) Of course, they do that it’s not about the buzz, but oftentimes that buzz is quite nice (laughs).” *(P17)

Some considered experiencing an altered state valuable in itself: realizing that unpleasant esketamine experiences passed away helped them recognize the transient nature of difficult experiences in daily life too. For others, the bizarreness of their experiences made them aware that these—like negative thoughts—are all created in their head, and that these experiences dissipate too.

*“It did teach me that* you *are everything (…) everything I think is inside my head. I experience all kinds of things [during the esketamine session]. Nothing happens to my body but in my head, I go through such bizarre journeys (…) And my head, that's me, that's everything. If I think in my head that I'm super, then I'm good. And if I think that I'm nothing, um then it* is *all nothing.”* (P4)

#### Mystical-type experiences

Several respondents described having experiences which took place in an infinite space, or outside of space and time. Experiences of ego dissolution, or disappearance of their sense of self, were closely related to unitive experiences, described as becoming part of something greater (the universe, the cosmos).

*“I can't describe it very well but (…) you realize what you are: I am the universe, or: I am Being. You step outside of your own specific little personhood; you get a kind of broader perspective on what you are, on what others are. This feeling of unity was very strong. So the person that you are necessarily loses its importance a bit.”* (P8)

The broader, cosmic perspective of being a tiny part of something larger than themselves also communicated a sense of relativeness or insignificance for some. Some respondents shared that the experience of relativeness contrasted strongly with the experience of being depressed and helped put their own problems into perspective.

*“Depression is (…) a kind of being trapped, (…) it's something very narrow. And that sense of unity is just something very wide, that opens you up, outwards, so [it’s] actually the opposite of depression, which is very much inward oriented.”* (P8)

Irrespective of their religious orientation, several participants described having religious-like experiences. The following participant, who did not consider herself a religious person, had an experience she described as spiritual:

*“Suddenly, a phrase came into my head: ‘Absorbed into the All’. (…) I never speak in words about “The All”, I don't even know exactly how to imagine this. But a tremendous peace came over me.”* (P17)

Another respondent, an active protestant, described one of her experiences as heavenly.

*“I never had such a concrete conception of heaven before, it was always something abstract. (…) I encountered billions of souls, floating around freely, not as human beings but as spirits, kind of like birds that all flew past each other (…) It really gave me a serene peace.”* (P16)

#### Fear and anxiety

Participants’ lived experiences were highly variable in terms of valence as well: respondents described positive experiences, but the majority (also) had experiences that were frightening, stressful or anxious.

*“Sometimes I have a bit of a horror experience: I can't get out and I don't know how long it will last because time just doesn't exist. (…) At first I think: shit, did I take too much? And then it goes wrong, because it all goes so fast and at a certain point, I can't think anymore. Then I’m just in that darkness and I can't leave, and think: shit, this wasn't supposed to happen (…). It takes forever. It is such an unpleasant experience.”* (P4)

For some participants during the esketamine sessions their negative thoughts, ruminations, and suicidal thoughts were intensified instead of disrupted; these negative emotions often stayed with them after the acute effects had worn off.

*“[During the ketamine sessions] I tended to get inside myself even more [mimicking fetal position], like 'I'm not there, I don't want to be there and I'm not there'. Like if you occupy less space, you also exist less.”* (P3)

Some frightening experiences were characterized by chaos, disorientation, feeling as if they had lost all contact with reality, as if they had lost their mind, were dying, or feared that they would never again return to sanity or to their body.

*“All of a sudden, my mind is in all kinds of other dimensions (…) I can't find my body, it doesn't exist anymore, neither does my husband or other people. (…) I feel like a hamster in one of those wheels, and I run and run and run but nothing happens. I am stuck, I can't get out anymore, nothing else exists but me, alone in these dimensions. (…) I think I'm dead, because I can't return to my body, time is endless, time doesn't exist.”* (P4)

#### Variability

The intensity and content of participant experiences varied substantially: for many participants, higher doses led to more intense and occasionally extraordinary experiences, whereas others had (unexpectedly) intense effects with lower doses. For some respondents, every session had roughly the same content and intensity; experiences for others varied substantially between sessions in both intensity and content. Participants found it difficult to describe their experiences; because they had never experienced anything like it; because the experience was indescribable, paradoxical, or contradictory; or because memories of the experience tended to fade quickly after the effects wore off.

*“It was something I had never experienced before. (…) I find it very difficult to describe, but I was completely out of this world, (…) I could no longer just think ‘oh this is the ketamine, I'm just lying on a bed, this is a side effect’. I was totally lost in infinity.” *(P10)

### Post-session experiences

#### Feeling hungover and fatigued

Many participants described having unpleasant physical side-effects immediately after the esketamine session ended, summarized as ‘hungover’: nausea, dizziness, headaches, muscle aches, blurry vision, occasionally vomiting, and a generally feeling of lousiness. These effects generally wore off on the same day.

*“I was very tired, probably because of the enormous tension and my resistance to it. I also had enormous muscle pain (…) just a stiff body. I was a bit creaky the day after and the whole afternoon afterwards I just wasn't worth a damn. (…) When I had thrown up, the day after would also be completely ruined. (…) A sore belly and a terribly wrinkly feeling, so the day after was actually gone.”* (P6)

Fatigue and feeling exhausted immediately afterwards were also commonly reported. This tiredness sometimes lasted a couple of hours, sometimes the rest of the session day, and occasionally on the following day as well. Various participants described going to bed or needing sleep shortly after the session to recuperate. Others noted impaired sleep quality, either because they were unable to sleep (well) or because they had more vivid dreams or nightmares.

*“When I managed to let go, the trips were okay, only the dreams that followed are annoying. But yeah, I’m not there for sweaty feet, these are thoughts related to my depression. So those come to the foreground more. Especially [this trauma] that seems to bother me greatly. (…) [It makes me] want to stay awake because I know I'm about to get [nightmares].”* (P1)

#### Lifting the blanket: mood effects

Effects on participants’ mood in the hours and days after sessions varied substantially too. Some had no perceptible effect on their mood, whereas others noticed distinct improvements. Yet others stated that esketamine provided a baseline mood stability or neutrality.

*“Ketamine ensures a kind of basic feeling level of, how to call it, a kind of neutral feeling of well-being. So not gloomy nor overly cheerful but just a good starting point, so to say. I have the idea that I now function more like a normal person than without the ketamine.”* (P16)

Participants used different metaphors to express their experience of being depressed: heaviness, darkness, hopelessness, bleakness, psychic pain, a diminishing world, or being submersed in a swamp. For some, this ‘heavy blanket’ or ‘grey veil’ had mostly disappeared.

*“[I used to wake up] covered by this blanket. Nausea, a tight throat, and no lust for life. Really thinking (soft voice) ‘screw it all’. And then it would lift again, but now [after the esketamine] it's just not there.”* (P1)

This mood lifting or stabilizing effect was mostly temporary, lasting one to sometimes three or four days.

*“I feel like I'm lying under a very heavy blanket, and because of the ketamine (…) a corner is lifted and I can look at the light. So, then my mood does improve a little, and that usually lasts about a day and a half, and then it subsides.”* (P10)

When respondents noticed improvement, they often mentioned appreciating sensory input again, noticing a reviving of dulled senses, such as tasting, smelling, and enjoying food again, seeing sharper and in color instead of in grey, and feeling emotions and feelings return.

*“Actually [when I was severely depressed] I felt little (…) It was just dead, I had no feeling. (…) When I say this now, I feel very guilty about it. But if my daughter or husband had died, I don't even think I would have felt the sadness. (…) When I looked at my daughter, I didn't feel that I loved her. But when I look at her now, I think gee I love her so very much. And I feel it too.” *(P4)

Some participants mentioned hypersensitivity and increased irritability; possibly related to feeling more open and vulnerable, after having had the depressive veil lifted.

*“On some days, I just notice sensitivity to small sounds (…). There are also days that, having recovered after two hours, then I can just function normally. [But on bad days] the world is more overwhelming; I am more sensitive to stimuli. Like in a supermarket I can more easily become a bit panicky, overwhelmed.”* (P11)

The broadened perspective enabled several respondents to make different life choices, such as eating healthier, drinking less alcohol, and exercising more; others (re)gained a perspective about their life and future. Participants also described recapturing a sense of enjoyment, and having more space and energy to undertake activities, practice art or sports, having more creativity, picking up hobbies, and initiating contacts with friends again.

*“My head used to be constantly giving negative impulses (…) coming up with a thousand scenarios in my head why things would go wrong or whatever. Yeah, and that's just gone. And if it is there for a moment I can stop it, and then it actually stops. That is wonderful. (…) I can read a book now and I can listen to my wife better. You know, there is room again to ask someone how they are doing. (…) There was just no room for that. [Now I have] a desire to do things again that wasn't there before. (…) Now things have a purpose: to be happy. (…) I can't really remember ever feeling this way. It has always been survival and now it's not.”* (P15)

## Discussion

This is the first qualitative study examining the phenomenology of the (sub)acute drug effects of oral esketamine in patients with severe, treatment-resistant depression, extending earlier findings on the role of ‘set and setting’ (Breeksema, Niemeijer, et al., 2022b). Esketamine induced subjective effects that varied significantly in intensity, content, valence, and on mood, both between and within subjects.

A recent qualitative study on patients with alcohol use disorder who received 0.8mg/kg racemic IV ketamine with psychological support described remarkably similar experiences (Grabski et al., 2022; Mollaahmetoglu et al., 2021). Despite discrepancies in enantiomers, route of administration, bioavailability, dosage, degree of psychological support, and patient populations, these similarities demonstrate ostensibly characteristic psychoactive features of ketamine: perceptual changes (auditory, visual, and proprioceptive); detachment (from body, emotion, mind, self, and the world); and mystical or transcendental experiences. In our study, the themes of openness, stillness, and spaciousness fit all three categories and were associated with transient cessation of negative thoughts and feelings (see Fig. 1).

In this article we used ‘detachment’ to describe specific facets of the phenomenology of the ketamine experience, rather than the commonly used ‘dissociation’. We argue that the construct is both too broad and narrow to describe ketamine’s effects. Existing scales (such as the Clinician-Administered Dissociative States Scale; CADSS) to measure dissociation were developed to measure dissociative aspects of post-traumatic stress disorder (Bremner et al., 1998). Dissociation is prevalent in a range of mental disorders (Lyssenko et al., 2018), and this close relation to psychopathology means that associations with this term are mainly negative. Further, these only partially describe the phenomenology of ketamine and show no consistent relationships nor offer a therapeutic rationale related to antidepressant response (Chen et al., 2022; Grabski et al., 2020; Luckenbaugh et al., 2014; Mathai et al., 2020; Sos et al., 2013). A possible exception is the ’depersonalization’ subscale (but not ‘derealization’ or ‘amnesia’) of the CADSS (Niciu et al., 2018). Interestingly, detachment, or disconnection, has been called a core element of depression (Karp, 1997; Osler, 2022), and there are other similarities in how depressive and ketamine states are described, such as diminished affect; emptiness or void; and decreased self-importance (Kendler, 2016). Importantly, the valence and meaning attributed to these states differed significantly. The feelings of worthlessness, despair, hopelessness, or numbness that often permeated the lives of depressed patients was temporarily interrupted during esketamine sessions, which created an openness that enabled them to consider other perspectives and outlooks. Future studies should refine and operationalize detachment (e.g., to oneself, body, negative emotions and thoughts, external surroundings) in the context of antidepressant response, and how this disconnection may relate to increased connectedness.

Using a spatial metaphor: whereas patients’ minds occupied a small gloom-filled space during their depression, the temporary expansion of this mind space made everything (including thoughts, anxieties, concerns etc.) appear smaller in comparison. Such a shift in perspective was frightening to some, but (also) helpful in contextualizing their perceived problems and realizing their relative insignificance and transience. This mind-expansion also made it easier for patients to connect to their own feelings, to others, and the outside world: the antithesis of disconnection and isolation. Multiple participants in our study described the intrinsic value of temporarily being lifted out of their depressive state, echoing reports from other qualitative studies (Griffiths et al., 2021; Lascelles et al., 2019; Mollaahmetoglu et al., 2021; Sumner et al., 2021; van Schalkwyk et al., 2017). Some critics contend that this temporary ‘high’ should not be confused with an antidepressant effect (Horowitz and Moncrieff, 2021). However, this acute, positively experienced effect is thought to play a central role in both psychedelic and ketamine assisted therapy modalities (Dore et al., 2019; Mathai et al., 2022a). Further, in ketamine treatment acutely experienced anxiety correlates negatively with treatment outcomes (Aust et al., 2019), whereas acutely experienced happiness correlates with longer-term positive outcomes (Chen et al., 2020), although anxiety did not appear to affect treatment retention (Bahji et al., 2022). In fact, the temporary interruption of incessant negative cognitions and feelings (also described elsewhere, see e.g. (Griffiths et al., 2021; Mollaahmetoglu et al., 2021)) makes ketamine suitable as a treatment for mood disorders, suicidal ideation and potentially for other mental disorders characterized by psychological rigidity (Servaas et al., 2021).

For some, this transient interruption created a window of opportunity associated with increased openness to engage in psychotherapy. Ketamine has been combined with mindfulness-based interventions (see (Payne et al., 2021) for further potential synergies) to treat substance use disorders (SUD) and PTSD (Azhari et al., 2021; Dakwar et al., 2019; Grabski et al., 2022; Pradhan et al., 2017; Pradhan and Rossi, 2020; Stocker et al., 2022), as ketamine-assisted psychotherapy or ketamine psychedelic therapy (Dore et al., 2019; Krupitsky and Grinenko, 1997) or with adjunctive cognitive-behavioral therapy (Wilkinson et al., 2017, 2021), favored by some experts as the current gold-standard paradigms (including third-wave therapies such as dialectical behavior therapy (DBT) and acceptance and commitment therapy (ACT)) (Yaden et al., 2022). Future studies should investigate whether combinations with psychotherapies can extend the therapeutic benefit of esketamine, to explore the optimal timing of such interventions (Mathai et al., 2022a), and look at appropriate modalities for TRD treatment.

Our respondents reported various elements of mystical-type experiences (ME) (MacLean et al., 2012); ME correlate positively with treatment benefits in different patient populations treated with serotonergic psychedelics (Johnson et al., 2019; Ko et al., 2022) and ketamine in SUD patients (Dakwar et al., 2014, 2018; Mollaahmetoglu et al., 2021; Rothberg et al., 2020; Sumner et al., 2021). Participants in our study also described these experiences as meaningful; future studies should systematically investigate to what degree ME mediate therapeutic outcomes in TRD treatment and explore appropriate therapeutic frameworks. Existing psychometric instruments may be suitable (e.g., MEQ-30 (Barrett et al., 2015; MacLean et al., 2012), 5D-ASC (Studerus et al., 2010), EDI (Nour et al., 2016), and WCS (Watts et al., 2022)), but a novel questionnaire that captures the unique profile of the acute effects of ketamine is needed.

Many participants reported moments of psychological distress. Common fears were related to experiences of detachment, e.g., being afraid of dying, of going crazy, or never returning to their body or reality. Dissociative effects and associated anxiety (e.g. paranoia, grief, panic, nausea) experienced even by healthy volunteers have been described as early as 1965 (Domino et al., 1965) and continue to be described today (Griffiths et al., 2021; Lascelles et al., 2019; Mollaahmetoglu et al., 2021; Sumner et al., 2021). This firmly underlines the need for continued emotional and psychological support throughout all stages of ketamine treatment, which is particularly crucial in vulnerable patients. Providing treatments that induce potentially destabilizing experiences without providing optimal preparation and continued support is both unethical and likely clinically inefficient, as transient fear and anxiety can exacerbate feelings of helplessness and pointlessness (Breeksema, Niemeijer, et al., 2022a). An important open question is whether these transient challenging experiences can be therapeutically beneficial, as has been suggested for classical psychedelics and entactogens (Barrett et al., 2016; Breeksema, Kuin, et al., 2022b). At minimum, proper support for ketamine treatment should consider the role of education and preparation for the variable and unpredictable states induced by ketamine; expectancy and expectations management; instructing calming techniques; creation of trust and rapport with an empathetic, caring and present staff; inclusion of trusted others throughout the process; and a treatment setting optimized for patient comfort (Breeksema, Niemeijer, et al., 2022b; Carhart-Harris et al., 2018; Eisner, 1997; Johnson et al., 2008). An important goal of intervention design should be to increase the likelihood of positively experienced sessions, to reduce occurrence and impact of anxious and fearful sessions, and ideally to also facilitate the safe emergence and integration of potentially salient psychological content (Dakwar et al., 2014). Anxiety can be mitigated by providing adequate education and preparation, psychological and emotional support, the use of music, and interpersonal rapport (Breeksema, Niemeijer, et al., 2022a; Ceban et al., 2021; Krupitsky et al., 2007); in case of extreme anxiety (low) doses of anxiolytic medication may alleviate patients’ distress (for an overview of potential interactions see (Veraart et al., 2021)).

### Strengths and limitations

A major strength of this study is the use of phenomenological methods, which allowed us to explore patients’ lived experiences of orally administered esketamine in far greater detail than standardized approaches. Asking open-ended questions enabled us to explore experiences and topics not covered in standardized questionnaires, not only generating a better understanding of beneficial and negative facets of orally administered esketamine, but also yielding insights into the diversity of psychological experiences that potentially mediate the therapeutic benefits of esketamine.

This study was conducted on oral esketamine for patients with severely treatment-resistant depression that may not be representative of large study populations; they are however probably representative of patients in real world settings seeking ketamine treatment. We also do not know whether other administrations and formulations induce similar effects, although comparison of our study with previous qualitative research does point in that direction. There is a potential risk of selection bias as we only interviewed patients who finished their initial 6-week treatment, although this was likely mitigated by the mix of positive and negative experiences reported by participants. The sample size could also raise questions about (external) validity although 17 respondents is a considerable sample size in phenomenological research.

Lastly, the therapeutic effects determined in our study were not assessed systematically. Lacking a therapeutic framework within which to understand and harness these effects, it remains to be seen whether these can indeed be used to optimize the therapeutic efficacy of esketamine. An important future step would be to explore whether the antidepressant response, psychological safety, and patient comfort can be enhanced by optimizing the treatment setting, and the degree of psychological support, and/or specific (psycho)therapeutic approaches.

## Conclusion

Oral esketamine induced a number of subjective effects that were highly variable in terms of intensity, content, valence, and impact on patients’ mood. Despite the regular pharmacological treatment context, in which patients merely received information about possible side effects and minimal preparation for the acute subjective effects, patients reported various effects that suggest a therapeutic potential for depression treatment, such as increased openness, detachment, interruption of negativity, and mystical-type experiences. Harnessing these distinctive properties of ketamine might provide an important opportunity to optimize or extend its antidepressant effects. Psychological distress was common in most patients, highlighting the need for ongoing emotional and psychological support in all stages of esketamine treatment and an optimized treatment setting.

## Data Availability

The data that support the findings of this study are not publicly available due to reasons of privacy of the participants and data protection regulations. Data may be made accessible upon request and after consultation with the data managing officers from the University Medical Center Groningen or by contacting the corresponding author.
